# Association between age of paediatric index cases and household SARS-CoV-2 transmission

**DOI:** 10.1017/S0950268824000918

**Published:** 2024-11-20

**Authors:** Amanda Markelz, Zachary Zirnhelt, Keeley Morris, Scott A. Seys, Abbey Ruhland, Ashley Fell, Lydia Fess, Kathryn Como-Sabetti, Stephanie Meyer

**Affiliations:** Infectious Disease Epidemiology, Prevention and Control (IDEPC) division at the Minnesota Department of Health, Saint Paul, MN, USA

**Keywords:** COVID-19, households, paediatric, regression analysis, SARS-CoV-2 transmission

## Abstract

SARS-CoV-2 transmission dynamics within households involving children are complex. We examined the association between paediatric index case (PIC) age and subsequent household SARS-CoV-2 transmission among cases reported to the Minnesota Department of Health between March 2021 and February 2022. In our primary analysis, we used logistic regression to estimate odds ratios adjusted for race/ethnicity, sex, geographic region, and disease severity among households with an unvaccinated PIC. We performed a secondary analysis among households where the PIC was eligible for vaccination adjusting for the same covariates plus time since the last vaccination. Both analyses were stratified by variant wave. During the Alpha wave, PICs of all age groups had similar odds of subsequent transmission. During Delta and Omicron waves, PICs aged 16–17 had higher odds of subsequent transmission than PICs aged 0–4 (Delta OR, 1.32; [95% CI, 1.16–1.51], Omicron OR, 4.21; [95% CI, 3.25–5.45]). In the secondary analysis, unvaccinated PICs had higher odds of subsequent transmission than vaccinated PICs (Delta OR 2.89 [95% CI, 2.18–3.84], Omicron OR 1.35 [95% CI, 1.21–1.50]). Enhanced preventative measures, especially for 12–17-year-olds, may limit SARS-CoV-2 transmission within households involving children.

## Introduction

Estimating household transmission dynamics of SARS-CoV-2, the virus that causes COVID-19 disease, is beneficial for identifying proper preventative measures, especially with the emergence of variants with varying degrees of transmissibility [1]. There is a lack of studies assessing household transmission of SARS-CoV-2 in children and adolescents. While children are less likely to experience severe health outcomes, they are not immune and may play an important role in disease transmission [1]. Although adults make up the largest proportion of total reported COVID-19 cases, the proportion of infected children has notably increased since the start of the pandemic [1, 2].

It is unclear to what extent children can transmit disease to other household members [3]. Several studies of household SARS-CoV-2 transmission assess secondary attack rates among household contacts [1, 3–6]. Fewer studies examine household transmission using regression analysis, which offers the benefits of adjusting for variables, reducing bias, and providing exponentiated coefficients that can be interpreted as odds ratios. [7–11].

Paul et al. determined that young children were more likely to be a source of household transmission than adolescent and teen index cases [7]. However, based on the timing of the study, the researchers were unable to address potential variations in household transmission during subsequent SARS-CoV-2 variant waves of Alpha, Delta, and Omicron. The Alpha variant was believed to be approximately 30–50% more contagious than the original SARS-CoV-2 strain, Delta was estimated to be 80–90% more transmissible than Alpha, and Omicron was more transmissible than Delta [12]. A study from Denmark estimated that the effective reproduction number of Omicron is 3.19 times greater than that of Delta [13].

Additionally, since the Paul et al. study, vaccines have become more widely available to paediatric groups as young as 6 months of age. There is a need to understand how vaccination impacts household transmission, especially among paediatric age groups. While some studies included vaccinated participants, some models could not adjust for vaccination status because not all participants were eligible for vaccination during the study period [8, 9].

Our primary analysis examined whether there was an association between the age of unvaccinated paediatric index cases (PICs) aged 0–17 years and household transmission of SARS-CoV-2 within three variants of concern (VOC) waves. Additionally, a secondary analysis examined how vaccination among paediatric groups impacted household transmission during VOC waves when children were eligible for vaccination. Both conducted analyses followed Strengthening the Reporting of Observational Studies in Epidemiology (STROBE) reporting guidelines.

## Methods

Healthcare facilities and laboratories are mandated to report information about all Minnesota residents who test positive for COVID-19 via PCR or antigen test to the Minnesota Department of Health (MDH). During the period of interest, individuals who tested positive were contacted and interviewed to gather data on various aspects including the date of symptom onset, race and ethnicity, residential living setting, hospitalization history, employment status, school or daycare affiliations, and participation in sports activities. Testing, demographic, and interview data are entered into the Minnesota Electronic Disease Surveillance System (MEDSS). Over 900 000 COVID-19 laboratory-confirmed cases were reported to MDH from March 2021 to February 2022.

COVID-19 cases were stratified using the specimen collection date into three VOC waves. A VOC wave was defined as consecutive weeks when a VOC represented greater than or equal to 50% of samples sequenced successfully among Minnesota residents. Alpha, Delta, and Omicron VOC waves included cases with specimen collection dates from 15 March through 15 June 2021, 15 August through 15 November 2021, and 1 January through 15 February 2022, respectively.

**Figure 1.**
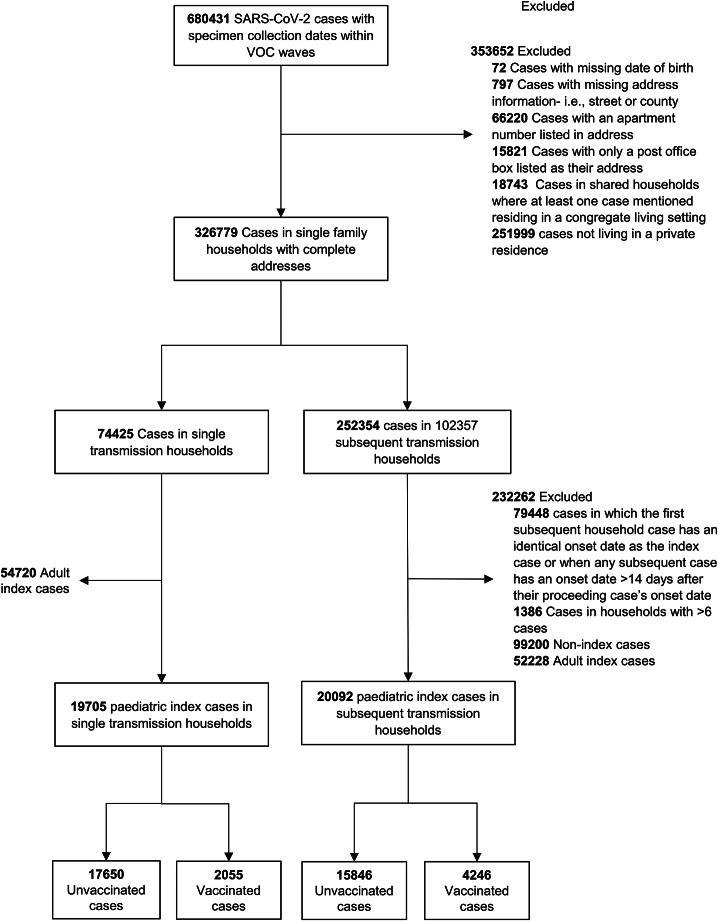
Flow diagram of study cohort for primary analysis.

A total of 680 431 cases occurred within the VOC waves included in this analysis (Figure 1). Case addresses were standardized and any cases missing date of birth (*n* = 72), street address or county (*n* = 797), and cases residing in multiunit apartment buildings (*n* = 66 220), congregate (*n* = 18 743), or non-private living settings (*n* = 251 999) were excluded (Figure 1). Cases were linked through deterministic data matching, a method that established exact or highly probable matches based on the street address and county information.

Cases were classified as living in single or subsequent transmission households. A single transmission household was defined as a private residence with a single laboratory-confirmed COVID-19 case reported at an address, while a subsequent transmission household was a private residence with more than one COVID-19 case reported at the same address. Index cases were defined as the first case in household based on the earliest COVID-19 symptom onset date. If the symptom onset date was missing or if the case was asymptomatic, the specimen collection date was used as a proxy; there were no instances where both dates were missing. If we could not identify an index case due to two cases within the household having the same onset dates, the household was excluded from the study.

Subsequent cases were defined as cases who had an onset date or specimen collection date 1–14 days after the index case. Subsequent transmission households with more than six cases (*n* = 1 386) were excluded to prevent the possibility of including a multiunit residence. Households were only counted once within a wave, therefore in any household with more than one transmission event within a single VOC wave, only the first event was included. It is possible that a household could be included in another VOC wave if a reinfection or other transmission event occurred. Reinfections were defined as a positive laboratory test (PCR or antigen) greater than 90 days from the previous positive test. Households where the index case could not be determined and households with adult index cases were removed as this analysis was kept to PICs only.

Two stratified analyses were performed. The primary outcome of interest was subsequent household SARS-CoV-2 transmission following a PIC. In the first analysis, we examined the association between the age of unvaccinated PICs and the occurrence of subsequent household transmission of SARS-CoV-2. In the secondary analysis, we explored the impact of vaccination on household transmission of SARS-CoV-2. Notably, this secondary analysis exclusively included paediatric age groups eligible for vaccination during the Delta and Omicron variant waves.

Age groups for PICs were classified mirroring vaccination eligibility groupings: 0–4, 5–11, 12–15, and 16–17 years old. Other surveillance data collected from MEDSS case reports and interviews included but were not limited to race/ethnicity, sex, geographical region, and vaccination status. Vaccination status was obtained from the Minnesota Immunization Information Connection (MIIC). The standard definition of a post-vaccination SARS-CoV-2 infection was used and defined as a laboratory-confirmed case (PCR or antigen) that occurred greater than or equal to 14 days post-completion of their primary vaccination series. We designated the fully vaccinated status to individuals who received a primary series with an FDA-approved vaccine; either one dose of the Janssen (Johnson & Johnson) vaccine or two doses of the Pfizer, Moderna, or Novavax vaccines.

## Statistical analysis

PICs within the three VOC waves were characterized using descriptive analyses. Characterization included breakdown by age group, geographic region, sex, race/ethnicity, and disease severity. Disease severity was defined as cases that were hospitalized and/or died. We further described cases that were and were not associated with subsequent household transmission. We used a multivariable logistic regression model adjusting for race/ethnicity, sex, geographic region (i.e., metropolitan vs. rural), and disease severity.

To account for the possibility that subsequent cases occurring very close to the same time as the household index case may have acquired their infection at the same time or even before the index case, we performed sensitivity analyses that examined whether the results remained consistent when subsequent cases were defined as occurring 2–14 or 4–14 days after the index case respectively. The same multivariable regression model was applied after reclassifying subsequent household cases using these new definitions.

Although paediatric vaccination eligibility was not uniform across different VOC waves, it is important to recognize that vaccination may have a significant impact on COVID-19 transmission. A secondary analysis including vaccinated PICs was performed to assess the impact of vaccination on the association of paediatric age and household transmission. Due to varying vaccination eligibility, only PICs aged 12 years and older were included in the Delta wave analysis and PICs aged 5 years and older were included in the Omicron wave analysis. We ran a similar multivariable logistic regression model adjusting for race/ethnicity, sex, geographic region, disease severity, and time since the last vaccination. All analyses were performed using SAS 9.4 (SAS Institute).

## Results

From the Alpha, Delta, and Omicron VOC waves, a total of 39 797 vaccinated and unvaccinated PICs from private residences were identified (Figure 1). The study population for the primary analysis included 9 476 unvaccinated PICs in the Alpha period, 16 001 in Delta, and 9 496 in Omicron (Table 1). The study population for the secondary analysis included 5 199 PICs ages 12–17 years and 8 555 PICs ages 5–17 years in the Delta and Omicron waves respectively (Table 2).

**Table 1.** Primary analysis: Characterization of paediatric index cases by association with subsequent household transmission by variant of concern wave

	Alpha, Index cases no. (%)		Delta, Index cases no. (%)		Omicron, Index cases no. (%)	
	Not associated with subsequent cases in the HH	Associated with subsequent cases in the HH	Not Associated with subsequent cases in the HH	Associated with subsequent cases in the HH	Not associated with subsequent cases in the HH	Associated with subsequent cases in the HH
Total no.	6 811	2 665	8 285	7 716	4 031	5 465
Age group						
0–4	903 (13.2)	363 (13.6)	1 549 (17.5)	1 313 (16.5)	2 385 (59.2)	2 599 (47.6)
5–11	1 996 (29.3)	797 (29.9)	4 668 (56.3)	4 040 (52.4)	1 383 (34.3)	1 861 (34.0)
12–15	2 387 (35.0)	941 (35.3)	1 430 (17.3)	1 626 (21.1)	184 (4.6)	652 (11.9)
16–17	1 525 (22.4)	564 (21.2)	638 (7.7)	737 (9.6)	79 (2.0)	353 (6.5)
County region, Metropolitan	3 697 (54.3)	1 534 (57.6)	4 299 (51.9)	3 605 (46.7)	2 437 (60.5)	3 196 (58.5)
Sex, Male	3 367 (49.4)	1 380 (51.8)	4 072 (49.2)	3 843 (49.8)	2 047 (50.8)	2 758 (50.5)
Severe disease	7 (1.1)	17 (0.6)	99 (1.2)	37 (0.5)	133 (3.3)	47 (0.9)
Race/Ethnicity						
White, non-Hispanic	4 884 (71.7)	1 866 (70.0)	6 801 (70.0)	5 093 (66.0)	2 424 (60.1)	3 005 (55.0)
Black, non-Hispanic	388 (5.7)	152 (5.7)	497 (6.0)	344 (4.5)	301 (7.5)	291 (5.3)
Asian, non-Hispanic	193 (2.8)	74 (2.8)	293 (3.5)	235 (3.1)	244 (6.0)	243 (4.5)
American Indian/Alaskan Native, non-Hispanic	99 (1.5)	19 (0.7)	149 (1.8)	119 (1.5)	56 (1.4)	58 (1.1)
Native Hawaiian/Pacific Islander, non-Hispanic	4 (0.1)	1 (0.1)	10 (0.1)	11 (0.2)	3 (0.1)	6 (0.1)
Multiple races, non-Hispanic	301 (4.4)	122 (4.6)	519 (6.3)	274 (3.6)	294 (7.3)	200 (3.7)
Other, non-Hispanic	75 (1.1)	31 (1.1)	73 (0.9)	78 (1.0)	47 (1.2)	81(1.5)
Hispanic	278 (4.1)	192 (7.2)	311 (3.6)	1 051 (13.6)	223 (5.5)	1 108 (20.3)
Unknown/missing	589 (8.6)	208 (7.8)	632 (7.6)	511 (6.6)	439 (10.9)	473 (8.7)

**Table 2.** Secondary analysis: Characterization of paediatric index cases eligible for vaccination by association with subsequent household transmission by variant of concern wave

	Delta, Index cases no. (%)		Omicron, Index cases no. (%)	
	Not associated with subsequent cases in the HH	Associated with subsequent cases in the HH	Not associated with subsequent cases in the HH	Associated with subsequent cases in the HH
Total no.	2 623	2 576	3 138	5 417
Age group				
0–4	─	─	─	─
5–11	─	─	2 379 (75.8)	2 860 (52.8)
12–15	1 706 (65.0)	1 726 (67.0)	541 (17.2)	1 649 (30.4)
16–17	917 (35.0)	850 (33.0)	218 (7.0)	908 (16.8)
County region, Metropolitan	1 157 (44.1)	942 (36.6)	1 919 (61.2)	3 170 (58.5)
Sex, Male	1 255 (47.9)	1 234 (47.9)	1 551 (49.4)	2 616 (48.3)
Severe disease	39 (1.5)	11 (0.4)	79 (2.5)	18 (0.3)
Vaccination status				
Unvaccinated	2 004 (76.4)	2 332 (90.5)	1 363 (43.4)	2 450 (45.2)
Vaccinated <3 months	191 (7.3)	75 (2.9)	1 298 (41.4)	1 420 (26.2)
Vaccinated >3 months	428 (16.3)	169 (6.6)	477 (15.2)	1 546 (28.6)
Race/Ethnicity				
White, non-Hispanic	1 776 (67.7)	1 632 (63.4)	1 986 (63.3)	2 978 (55.0)
Black, non-Hispanic	199 (7.6)	132 (5.1)	194 (6.2)	300 (5.5)
Asian, non-Hispanic	78 (3.0)	57 (2.2)	196 (6.2)	284 (5.2)
American Indian/Alaskan Native, non-Hispanic	70 (2.7)	52 (2.0)	45 (1.4)	54 (1.0)
Native Hawaiian/Pacific Islander, non-Hispanic	2 (0.1)	4 (0.2)	2 (0.1)	9 (0.2)
Multiple races, non-Hispanic	128 (4.9)	69 (2.7)	203 (6.4)	171 (3.2)
Other, non-Hispanic	28 (1.0)	36 (1.4)	30 (1.0)	82 (1.5)
Hispanic	225 (8.6)	176 (6.8)	329 (10.5)	459 (8.5)
Unknown/missing	117 (4.4)	418 (16.2)	153 (4.9)	1 080 (19.9)

**Figure 2.**
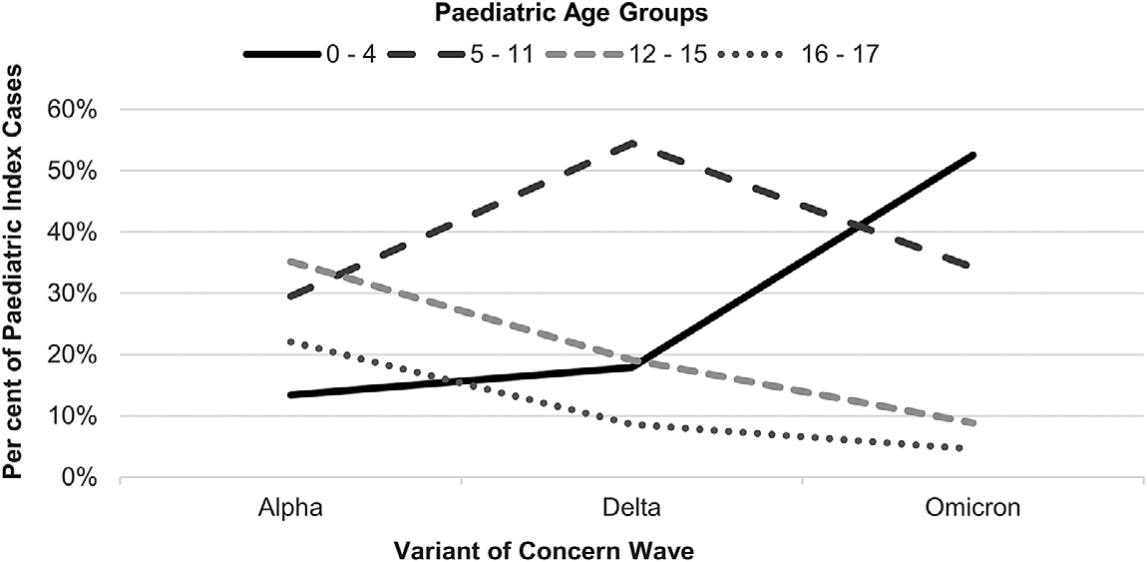
Age composition of unvaccinated of paediatric index cases by variant wave.

Overall, in Minnesota, across all three VOC waves, 15 846 (45.3%) households with an unvaccinated PIC experienced subsequent household transmission, including 2 665 (28.1%) households during the Alpha wave, 7 716 (48.2%) during the Delta wave, and 5 465 (57.6%) during the Omicron wave. The age composition of unvaccinated PICs varied by VOC wave (Figure 2). For example, during the Alpha wave, the highest proportion of PICs occurred among children ages 12–15 years (35%) and the lowest proportion of PIC’s were the 0–4-year age group (13%). Whereas during the Omicron wave, the highest proportion of PICs were among children ages 0–4 years (52%) and individuals ages 16–17 years accounted for the smallest proportion of PICs (5%). Characterization by age group, geographic region, sex, race/ethnicity, disease severity, and association with subsequent household transmission are shown in Table 1.

**Figure 3.**
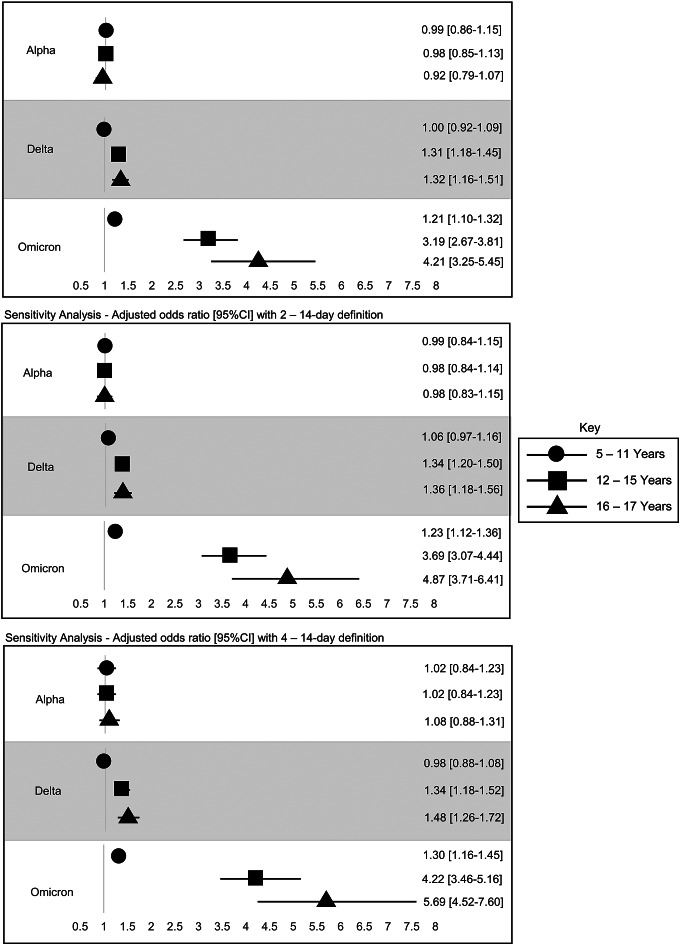
Adjusted odds ratios [95% CI] with 1–14-day definition compared to 0–4-year-old reference age group. Multivariable logistic regression models are adjusted for race/ethnicity, sex, geographic region, and disease severity.

During the Alpha wave, PICs of all age groups had similar odds of transmitting SARS-CoV-2 to a household member (Figure 3). During the Delta wave, PICs ages 16–17 (OR, 1.32 [95% CI 1.16–1.51]) and 12–15 (OR, 1.31 [95% CI 1.18–1.45]) years had higher odds of transmitting SARS-CoV-2 to a household member than PICs ages 0–4 years. During Omicron, PICs ages 16–17 (OR, 4.21 [95% CI 3.25–5.45]), 12–15 (OR, 3.19 [95% CI 2.67–3.81]), and 5–11 years (OR, 1.21 [95% CI 1.10–1.32]) had higher odds of transmitting SARS-CoV-2 to a household member than PICs ages 0–4 (Figure 3).

Sensitivity analyses using the same regression model with an alternative household transmission definition from 2–14 and 4–14 days showed that while the relationship among age group and subsequent household transmission remained consistent with the primary findings, odds ratios increased with both 2–14 and 4–14-day changes to the classification of subsequent cases (Figure 3).

## Household transmission by vaccination status

Characterization by age group, geographic region, sex, disease severity, vaccination status, race/ethnicity, and association with subsequent household transmission are shown in Table 2. During the Omicron wave, PICs ages 16–17 (OR, 3.73 [95% CI 3.16–4.41]) and ages 12–15 years (OR, 2.75 [95% CI 2.44–3.09]) had higher odds of transmitting SARS-CoV-2 to a household member than PICs ages 5–11 years after adjusting for overall vaccination status.

**Figure 4.**
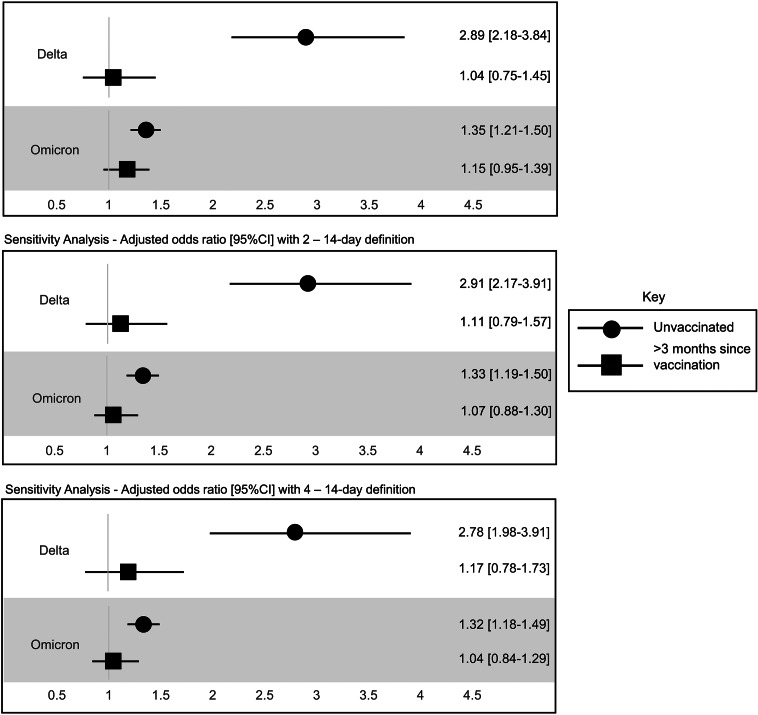
Adjusted odds ratios [95% CI] with 1–14-day definition compared to <3 months since vaccination reference group. Multivariable logistic regression models are adjusted for race/ethnicity, sex, geographic region, and disease severity.

A multivariable logistic regression model that adjusted for race/ethnicity, sex, geographic region, disease severity as well as time since the last vaccination, indicated that during the Delta wave PICs who were unvaccinated across 12–15 and 16–17-year-old age groups had 2.89 [95% CI 2.18–3.84] times the odds of subsequent household transmission than PICs vaccinated less than 3 months before infection (Figure 4). Those vaccinated more than 3 months before infection had similar odds of transmission as PICs vaccinated less than 3 months before infection (OR, 1.04 [95% CI 0.75–1.45]). During the Omicron wave PICs who were unvaccinated across 5–11, 12–15, and 16–17-year-old age groups had 1.35 [95% CI 1.21–1.50] times the odds of subsequent household transmission than PICs vaccinated less than 3 months before infection (Figure 4). Those vaccinated more than 3 months before infection had slightly higher odds of transmission than PICs vaccinated less than 3 months before infection, however, these results were not statistically significant (OR, 1.15 [95% CI 0.95–1.39]).

We conducted sensitivity analyses using the 2–14 and 4–14-day household transmission definitions using the model adjusting for time since the last vaccination. These analyses suggest that the relationship between vaccination status and subsequent household transmission remained consistent with the main study results independent of the definition of household transmission (Figure 4).

## Discussion

Our analysis suggests that in Minnesota during the Alpha wave, there may not have been a difference in subsequent household transmission across paediatric age groups. However, during the Delta and Omicron waves, infections among 5–11, 12–15, and 16–17-year-olds may have been more likely to result in at least one additional household case compared to PICs ages 0–4 years. Even though cases aged 16–17 years represented the smallest proportion of PICs and were eligible for vaccination prior to other paediatric ages (Figure 2), they appeared to be more likely to transmit SARS-CoV-2 to household contacts during Delta and Omicron waves even after adjusting for vaccination status.

There are several possible explanations for our results. Social and/or behavioural differences in masking, social distancing, and extracurricular activities may have varied across time and by age group [14]. These behavioural traits were not collected and could not be assessed in this analysis. Our results differed from previous findings which found that younger children may be more likely to transmit SARS-CoV-2 infection compared with older children, and the highest odds of transmission was observed for children aged 0–3 years [7]. A possible explanation for the difference in results may be the difference in timing. The previous study took place during an earlier time when schools were more likely to be using distance learning options, which may have reduced the number of contacts between school-aged children. In our analysis, schools in Minnesota were back in session with in-person learning during this timeframe. Significant transmission occurred through sports and extracurricular activities in school-aged children [15]. In addition, previous studies occurred during a time when different SARS-CoV-2 variants were circulating and before COVID-19 vaccines were available. The changes in transmissibility in different variants may play a role, particularly in combination with vaccination status or timing. Another possible reason for greater transmissibility across older paediatric age groups could simply be the volume of virus expelled during coughing or symptomatic presentation in older children [16, 17].

Our analysis has several limitations. First, at-home tests are not included in communicable disease reporting in Minnesota and could not be accounted for in our analysis. The use of at-home testing likely increased over the course of our analysis period and may have been more common within households that already had a known laboratory-confirmed case of COVID-19. This may have contributed to undercounting the number of cases in households and secondary transmission cases, especially during the Omicron wave when at-home testing was widely available. Additionally, since at-home tests are not approved for children under the age of 2 years [18], there may be differential use of at-home tests by age due to parents seeking healthcare for younger children versus older children even with similar symptoms. Secondly, asymptomatic cases are often missed by surveillance and can lead to undercounting cases. Households are likely biased towards those with more symptomatic children while the less symptomatic are less likely to be identified or seek testing [19]. Surveillance systems that solely detect symptomatic children will be unsuccessful in recognizing asymptomatic individuals who could still be spreading the virus in their community and schools [19]. Age may be associated with asymptomatic infections and that could potentially impact our results; however, symptom status does not necessarily correlate with viral load in children [19, 20]. Third, it is important to note that this study lacked sequencing data to definitively confirm the variant responsible for illness in the index case and to verify that any subsequent household member was truly infected by the index case.

Surveillance data does not include total household number or a full description of who resides in a case household. This limitation inhibited the ability to adjust for covariates such as the vaccination status of other household members and total household size. It is likely that vaccinated PICs reside in households with other household members who are also vaccinated, and this analysis could not distinguish whether reductions in secondary transmission are due to the vaccination status of the PIC or fellow household members. Additionally, covariates such as the number of household members susceptible to secondary transmission, including scenarios of overcrowding, could not be considered for the same reason. There is the potential that some cases we classified as subsequent transmission were in fact instances of concurrent exposure between the index case and subsequent cases. We addressed the potential for misclassification by running a sensitivity analysis to measure the effect of changing the definition of subsequent household transmission to more conservative options. These options resulted in greater differences in the odds of subsequent household transmission from a 16–17-year-old PIC than from a 0–4-year-old PIC. We relied on reported street address and county in the state-based case reporting system which may have resulted in misclassification of household members based on similar or incorrect addresses. This could have also led to misclassification of households and does not account for an individual having multiple households (e.g., blended families). In addition, we did not account for variations in COVID-19 mitigation practices such as mask use, business capacity, childcare requirements, and restrictions, which may limit generalizability to populations with different behavioural practices.

Benefits of this analysis include the use of a large and representative sample of Minnesota residents in addition to a secondary analysis that provides insight on the impact of vaccination on household transmission. Our analysis was stratified by vaccination status, which was done to ensure that the assumption of positivity was not violated, meaning that every participant had a positive chance of being included in the exposure (vaccinated) group. This approach was necessary because not all participants were eligible for vaccination during the study period. Overall, unvaccinated PICs in our analysis were more likely to transmit SARS-CoV-2 to a household member, but the odds ratios of secondary transmission from an unvaccinated PIC compared to a vaccinated PIC decreased noticeably during the Omicron wave compared to the Delta wave suggesting that COVID-19 vaccines may have been less protective against household transmission during the Omicron wave than the Delta wave. These results suggest that independent of vaccination status, 16–17 and 12–15-year-old PICs were more likely to transmit SARS-CoV-2 to a household member than younger children. Additionally, there were no significant differences in odds of household transmission in PICs that were infected more than 3 months from their last vaccination and PIC infected less than 3 months from their last vaccination.

Young children and adolescents play a complex role in the dynamics of household transmission. Regression analysis is beneficial to understand the association of predictor variables, such as race/ethnicity, geographic region, disease severity, and vaccination status, on the outcome of interest and can provide a framework for improved understanding of household transmission dynamics of SARS-CoV-2 [10, 11]. Our analysis adds to prior knowledge by assessing household SARS-CoV-2 transmission among PICs across multiple variant waves and stratifying by vaccination status. As SARS-CoV-2 continues to evolve and more paediatric age groups become eligible for vaccination and additional doses, it will be important to understand how the relationship between variants and COVID-19 vaccination affects the dynamics of household transmission. Public health initiatives and education that are targeted specifically to adolescents such as social media campaigns to enhance COVID-19 knowledge and promote preventive measures like hand hygiene can be effective [21]. Public health professionals should advocate for supportive environments for informed decision-making and encourage open discussions among family and peers about COVID-19 [21]. Furthermore, advocating for regular COVID-19 testing for individuals experiencing respiratory symptoms enhances early detection and containment efforts which is important for curbing transmission within households and communities.

Although our analysis offers suggestive evidence regarding the effect of age on household SARS-CoV-2 transmission, it is important to note that these conclusions may not be definitive due to the limitations of the available data. The focus for future studies might include the association of behavioural factors such as masking, test-seeking, or the ability to stay away from others while actively ill. Studies that specifically enumerate household characteristics including the total number of people living in the household, age, and vaccination status of household members would also be beneficial.

## Data Availability

The data that support the findings of this study are available on request from the corresponding author. The data are not publicly available due to case-patient data reported to MDH that is used in this analysis and is considered private information; we are unable to post to an open public repository.

## Abbreviations

PIC: paediatric index case
VOC: variant of concern
